# EGCG Alleviates DSS-Induced Colitis by Inhibiting Ferroptosis Through the Activation of the Nrf2-GPX4 Pathway and Enhancing Iron Metabolism

**DOI:** 10.3390/nu17030547

**Published:** 2025-01-31

**Authors:** Junzhou Chen, Conghui Yin, Yilong Zhang, Xin Lai, Chen Liu, Yuheng Luo, Junqiu Luo, Jun He, Bing Yu, Quyuan Wang, Huifen Wang, Daiwen Chen, Aimin Wu

**Affiliations:** 1 Institute of Animal Nutrition, Sichuan Agricultural University, Chengdu 611130, China; chenjunzhou2023@163.com (J.C.); 18735193091@163.com (C.Y.); 18563633520@163.com (Y.Z.); xin.lai2018@gmail.com (X.L.); liuchen-ipp@hotmail.com (C.L.); luoluo212@126.com (Y.L.); 13910@sicau.edu.cn (J.L.); hejun8067@163.com (J.H.); ybingtian@163.com (B.Y.); ice_5885327@163.com (Q.W.); wanghuifen1005@163.com (H.W.); 2 Key Laboratory for Animal Disease-Resistance Nutrition, China Ministry of Education, Sichuan Agricultural University, Chengdu 611130, China

**Keywords:** colitis, ferroptosis, iron metabolism, Epigallocatechin-3-Gallate (EGCG), antioxidant

## Abstract

Background: Ferroptosis is a regulated cell death process linked to various diseases. This study explored whether Epigallocatechin-3-gallate (EGCG), a tea-derived antioxidant, could regulate ferroptosis to alleviate dextran sulfate sodium (DSS)-induced colitis. Methods: A DSS-induced colitis model was used to assess EGCG’s effects. Ferroptosis markers, oxidative stress, and iron metabolism were evaluated, alongside Nrf2-GPX4 pathway activation and ferritin (FTH/L) expression. Results: Iron dysregulation and oxidative stress contributed to DSS-induced colitis by activating ferroptosis in colonic epithelial cells. EGCG supplementation inhibited ferroptosis, reducing oxidative damage. Mechanistically, EGCG activated the Nrf2-GPX4 pathway, enhancing antioxidant defense, and improved iron metabolism by upregulating ferritin expression. Conclusions: EGCG effectively suppressed DSS-induced ferroptosis and colitis, highlighting its potential as a ferroptosis inhibitor and therapeutic agent.

## 1. Introduction

Inflammatory bowel disease (IBD), including Crohn’s disease and ulcerative colitis, is a chronic inflammatory disorder of the gastrointestinal tract that has become increasingly prevalent worldwide [1]. In recent decades, the incidence of IBD has risen significantly in developed countries and is now emerging as a major public health challenge in developing nations [2]. Patients with IBD suffer from recurring episodes of abdominal pain, diarrhea, weight loss, and malnutrition, often leading to a substantial decline in quality of life [3]. The pathogenesis of IBD is complex, involving genetic predisposition, environmental factors, gut microbiota dysbiosis, and immune system dysregulation [4]. Furthermore, IBD is associated with an elevated risk of colorectal cancer and poses a considerable burden on healthcare systems [5]. Consequently, understanding the underlying mechanisms of IBD and identifying effective therapeutic strategies remain critical areas of research.

Recent studies have highlighted the importance of oxidative stress and iron metabolism dysregulation in the pathophysiology of IBD [6,7]. Iron is an essential trace element required for numerous physiological processes, including oxygen transport, DNA synthesis, and energy metabolism [8,9]. However, disruptions in iron homeostasis, particularly the accumulation of excess-free iron, can trigger oxidative stress and lipid peroxidation through the Fenton reaction [10,11]. These processes contribute to cellular damage and death, exacerbating intestinal barrier dysfunction and inflammation.

Ferroptosis is a distinct form of regulated cell death characterized by iron-dependent lipid peroxide accumulation, which causes oxidative stress and cell membrane damage [12]. Unlike other forms of cell death such as apoptosis and necrosis, ferroptosis involves a unique biochemical pathway driven by excessive iron levels and the associated oxidative stress [13]. This process begins with the Fenton reaction, where iron catalyzes the formation of reactive oxygen species (ROS), particularly hydroxyl radicals, that attack unsaturated lipids in cell membranes, leading to lipid peroxidation [14]. This oxidative damage disrupts cell membrane integrity, ultimately resulting in cell death [14,15]. The regulation of ferroptosis involves a delicate balance between iron metabolism, antioxidant defenses, and lipid peroxidation processes [16]. Key players in this regulatory network include glutathione peroxidase 4 (GPX4), which detoxifies lipid peroxides by converting them into less harmful alcohols, and solute carrier family 7 member 11 (SLC7A11), which imports cystine to support glutathione synthesis, a critical antioxidant in cells [17]. When GPX4 is inhibited or when glutathione levels are depleted, lipid peroxides accumulate, and ferroptosis is triggered [17,18]. This process has been implicated in several pathological conditions, including cancer, neurodegenerative diseases, and inflammatory disorders like colitis [19,20]. In the context of IBD, ferroptosis is closely associated with intestinal epithelial cell damage and gut barrier dysfunction. Studies have shown that IBD patients exhibit elevated iron levels in the gut, possibly due to increased iron absorption and impaired barrier integrity [21]. Excess-free iron promotes oxidative stress, lipid peroxidation, and inflammatory responses, creating a vicious cycle of tissue damage and chronic inflammation [13,22]. Additionally, ferroptosis may disrupt the gut microenvironment, perpetuating inflammation and disease progression. These findings suggest that targeting ferroptosis could provide a novel therapeutic approach for IBD.

Epigallocatechin-3-gallate (EGCG), a major polyphenolic compound in tea leaves, has garnered significant attention for its potent antioxidant and iron-chelating properties [23]. EGCG can bind to free iron, preventing its involvement in the Fenton reaction, thereby reducing the generation of ROS and lipid peroxidation [24,25]. Additionally, EGCG modulates the expression of key ferroptosis regulators, including glutathione peroxidase 4 (GPX4) and solute carrier family 7 member 11 (SLC7A11), and it upregulates ferritin heavy chain/light chain (FTH/L), facilitating the sequestration of excess-free iron [26]. These mechanisms collectively mitigate ferroptosis and protect cells from oxidative damage [27]. Several studies have demonstrated that EGCG can alleviate inflammation and oxidative stress in colitis, highlighting its potential therapeutic benefits in inflammatory bowel disease (IBD) [28]. However, the exact mechanisms underlying these effects remain poorly understood. While prior research has largely focused on EGCG’s antioxidant and anti-inflammatory properties, no studies have specifically addressed whether EGCG alleviates colitis by modulating ferroptosis, an iron-dependent form of cell death implicated in intestinal epithelial damage.

This study aims to investigate the protective effects of EGCG in DSS-induced colitis, focusing on its mechanisms in regulating ferroptosis and improving oxidative stress and iron metabolism. The results revealed that iron dysregulation and oxidative stress contributed to the activation of ferroptosis in colonic epithelial cells, which worsened colitis. EGCG supplementation suppressed ferroptosis by enhancing antioxidant defenses through the activation of the Nrf2-GPX4-signaling pathway and by improving iron metabolism via the upregulation of ferritin (FTH/L). By mitigating oxidative damage and regulating ferroptosis, EGCG effectively alleviated the symptoms of DSS-induced colitis. These findings suggest that EGCG holds promise as both a ferroptosis inhibitor and a therapeutic option for colitis.

## 2. Materials and Methods

### 2.1. Chemicals and Antibodies

EGCG (purity ≥ 98%) was provided by Teaturn Bio-pharmaceutical Co., Ltd. (Wuxi, China). Anti-TfR1, SLC40A1, FTH/L, Nrf2, pNrf2, GPX4, P62, and HO-1 were purchased from Abcam; anti-Keap1, SLC3A2, and SLC7A11 were purchased from Cell Signaling Technology; and β-actin was purchased from Santa Cruz biotechnology (Dallas, TX, USA).

### 2.2. Animal Experiment

The animal experimental procedure, approved by the Institutional Animal Care and Use Committee of Sichuan Agricultural University (SICAU-2023-082; date: 4 September 2023), involved 40 healthy 5-week-old C57BL/6N male mice housed in a controlled environment (24 ± 2 °C, 45–55% humidity, 12 h light/dark cycle) with ad libitum access to food and water. Mice were monitored daily for signs of distress (e.g., hunching, reduced movement, severe diarrhea), with humane endpoints defined as >20% body weight loss, severe dehydration, or persistent distress unresponsive to care. Inclusion criteria required healthy mice, while exclusion criteria involved signs of severe distress, >15% weight loss, or unrelated illness. Excluded mice were removed, and reasons were recorded.

**Figure 1:**
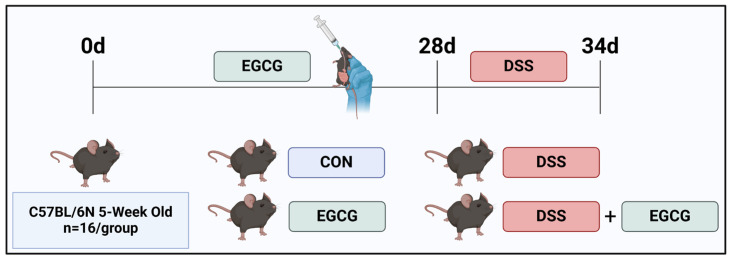
**Timeline of the animal experiment.** The test mice were divided into four groups as follows. Control group: PBS; EGCG group: 50 mg EGCG/kg body weight; DSS group: 3% DSS; DSS + EGCG group: 3% DSS + 50 mg EGCG/kg body weight. *n* = 16. The picture was created with BioRender.com (accessed on 10 March 2023).

All mice were randomly divided into a 2 × 2 factorial arrangement, fed irradiated basic diets, and gavaged with PBS or 50 mg EGCG/kg body weight per day, respectively (*n* = 16). The experiment lasted for 5 weeks, including a 4-week EGCG gavage and a 1-week DSS challenge period. DSS (2.5%, dextran sulfate sodium) was administered through drinking water to induce colitis. After the treatment, the animals were then anesthetized with isoflurane and subsequently euthanized (Figure 1).

To minimize potential confounders, all cages were rotated weekly within the animal housing facility to control for potential environmental effects, and feeding schedules were standardized to avoid circadian influences on metabolic outcomes.

### 2.3. Sample Collection

Blood was collected via cardiac puncture using anticoagulant tubes (approximately 2–3 drops). The remaining blood was transferred to 1.5 mL EP tubes and centrifuged at 4500 rpm for 10 min to collect serum. Approximately 30 mg of liver, spleen, and kidney tissues were placed into 1.5 mL EP tubes with tissue iron digestion solution for iron determination. The colon was divided into four sections, stored in separate 1.5 mL EP tubes, and used for analyses of iron content, western blot (WB), qPCR, and enzyme activity.

### 2.4. ROS and Lipid ROS Determination

To measure ROS, 50 μM H_2_DCFDA (Sigma–Aldrich, D6883, St. Louis, MO, USA) was added to mouse colonic epithelial cells (MCEC) and incubated for 30 min at 37 °C. Lipid ROS was then detected by staining the cells with 5 μM of C11-BODIPY (Thermofisher, Waltham, MA, USA, D3861) for 30 min at 37 °C. After washing the cells three times with PBS, flow cytometry was used to analyze the results. The flow cytometric analysis was performed using a BD FACSAria III flow cytometer (BD Biosciences, Franklin Lakes, NJ, USA). The samples were analyzed under the following conditions: excitation wavelengths of 488 nm for FITC, 640 nm for PE, and 405 nm for APC channels. Emission wavelengths were set at 530/30 nm for FITC, 585/42 nm for PE, and 670/30 nm for APC. Voltage settings for forward scatter (FSC) and side scatter (SSC) were optimized for each sample to ensure accurate signal detection.

### 2.5. Measurement of Fe^2+^

To measure the intracellular ferrous iron (Fe^2+^) level, mouse colonic epithelial cells were seeded into 12-well plates. When the cells reached about 70% of the plates, they were treated with different concentrations of Fe_3_S_4_ nanozymes (0, 25, 50, 100 μg/mL) for 24 h and then incubated with 1 μM of Far-Red Labile Fe^2+^ dye at 37 °C for 30 min without light. After washing with PBS, the intracellular Fe^2+^ levels were analyzed by flow cytometry.

### 2.6. Blood Parameter Determination

The following blood parameters were measured using an automatic biochemical analyzer (Beckman Coulter, München, Germany): red blood cell count (RBC), hemoglobin (HGB), hematocrit (HCT), mean corpuscular volume (MCV), and mean corpuscular hemoglobin (MCH). Blood samples were collected via cardiac puncture on the day of sampling to ensure accuracy. All measurements were performed according to the manufacturer’s instructions, and the assays were conducted in triplicate to ensure reliability.

### 2.7. Determination of Iron Parameters

Serum iron and non-heme iron contents in liver, spleen, and kidney tissues were determined. The measurement of liver, spleen, and kidney non-heme iron was performed as described previously by [29], with minor modifications. Briefly, tissues were homogenized in ice-cold saline, while non-heme iron was extracted using an acid digestion method and then measured by spectrophotometry. Serum iron, total iron-binding capacity (TIBC), unsaturated iron-binding capacity (UIBC), and serum transferrin (TF) were measured as described previously [30], with the addition of standard curve calibration for accurate quantification (Pointe Scientific, 416501-173). All assays were performed in triplicate.

### 2.8. Histological Examination of Colon

Colon tissue sections were collected from the same part (proximal colon) to minimize variability. The tissues were fixed in 4% buffered formalin at 4 °C for 24 h, then embedded in paraffin and sectioned into 4 μm-thick slices. Sections were stained with Hematoxylin and Eosin (HE) and Perls Prussian blue for evaluating tissue morphology and iron deposition, respectively. Histopathological changes were observed under a Zeiss Axioscope 5/7 Microscope.

### 2.9. Measurement of Malondialdehyde (MDA) and Glutathione (GSH) Levels

The levels of malondialdehyde (MDA) and glutathione (GSH) were measured using commercially available assay kits from Jiancheng Bioengineering Institute (Nanjing, China, GSH A003-1-2, MDA A003-4-1) following the manufacturer’s protocol. MDA, an indicator of lipid peroxidation, was determined using the thiobarbituric acid reactive substances (TBARS) assay, and GSH levels were assessed using a colorimetric assay. All measurements were performed in triplicate, and results were expressed as nanomoles (nmol) per milligram of tissue.

### 2.10. qPCR and Western Blot

The extraction of hepatic and splenic total RNA was conducted by TRIzol reagent (Invitrogen, Waltham, MA, USA, 108-95-2) according to the manufacturer’s specifications and diluted to 1 μg/uL. The working solution was configured in accordance with the protocols shown in the Reverse Transcription Kit (Takara, San Jose, CA, USA, RR037Q). The ΔΔCT method was used to calculate relative gene expressions by collecting the cycle threshold (Ct) and normalizing it to the housekeeping gene HPRT [31].

The isolation of colon total protein was performed with a RIPA buffer with phenylmethanesulfonylfluoride (PMSF, BeiJing, China, 1 mM). Western blotting was conducted as described previously [30]. The primary antibodies were diluted at 1:1000. CD98 (CST, 13180, Danvers, MA, USA), SLC7A11 (CST, 98051), pNRF2 (Abcam, ab76026, Cambridge, UK), NRF2 (Abcam, ab137550), HO-1 (Abcam; ab13248), P62 (Abcam, ab56416), Keap1 (Abcam, ab28913), GPX4 (Abcam, ab125066), TFR1 (Abcam, ab84036), Fpn (Abcam, ab78066), FTH/L (Abcam, ab75973). IL-6 (Abcam, ab68933), IL-1β (Abcam, ab387291), TNF-α (Abcam, ab478219), and ACSL4 (Abcam, ab512982). Secondary goat anti-mouse and anti-rabbit antibodies conjugated with HRP (Santa Cruz, Dallas, TX, USA sc-2030 and sc-2031) were diluted to a 1:3000 solution.

### 2.11. Enzyme-Linked Immunosorbent Assay (ELISA)

Serum IL-1β, TNF-α, and IL-8 contents were determined using the IL-1β (Jiangsu Meimian Industrial Co., Ltd., Cat#: MM-0905 M1, Nanjing, China), TNF-α (Jiangsu Meimian Industrial Co., Ltd., Cat#: MM-0132 M1), and IL-8 (Jiangsu Meimian Industrial Co., Ltd., Cat#: MM-0123 M1) ELISA kits, respectively. The actual operational steps were consistent with the instructions provided in the manual and were detailed in the Supplementary Materials.

### 2.12. Statistical Analysis

This study was conducted as a 2 × 2 two-factorial experiment. The results demonstrated the effects of iron overload and EGCG on mice and the interaction between high iron and EGCG in mice. All the data are expressed as the means ± standard errors (SEM) using Excel software, and we used the *t*-test and One-way/Two-way Analysis of Variance (ANOVA) to determine the relationships between groups [32]. Statistical significance levels were *p* < 0.05 (*), *p* < 0.01 (**), and *p* < 0.001 (***). All results were generated using GraphPad Prism 9 (GraphPad Software, San Diego, CA, USA).

Prior to statistical analyses, data normality and homogeneity of variance were assessed using Shapiro–Wilk and Levene’s tests, respectively. In cases where assumptions were violated, appropriate non-parametric tests or data transformations were applied.

### 2.13. Blinding

Blinding was implemented during the outcome assessment and data analysis stages. The investigator responsible for data collection and analysis was unaware of the group allocation to prevent potential bias.

## 3. Results

### 3.1. Preventive EGCG Supplementation Alleviates DSS-Induced Colitis by Reducing Inflammation and Improving Colon Morphology

**Figure 2:**
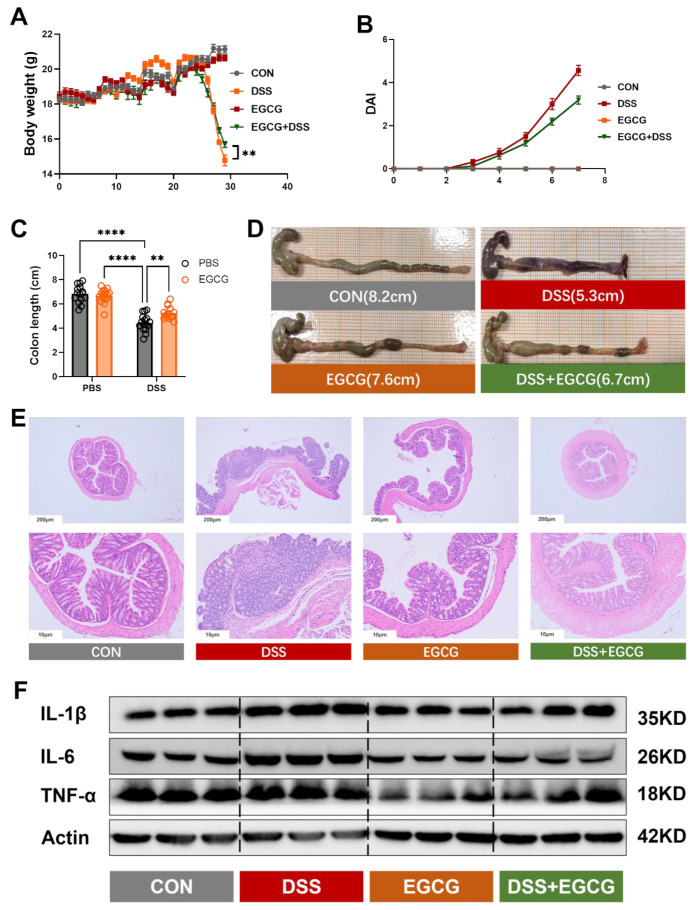
**EGCG significantly prevents growth impairment and intestinal inflammation caused by colitis.** (**A**) Body weight of mice in four groups was measured throughout the experiment. Mouse body weight was recorded every 2 days throughout the experiment (** *p* < 0.01) (*n* = 16). (**B**) The disease activity index in the last week among different treatment groups (*n* = 16). (**C**) Colon length (*n* = 16). (**D**) Measurement of colon length (*n* = 16). (**E**) Representative photographs of mice colon (scale bar = 200 μm and 50 μm) (*n* = 16). (**F**) The protein expression of IL-1β and TNF-α in colon (*n* = 3). Statistical significance is indicated as follows: ** *p* < 0.01, **** *p* < 0.0001.

To investigate the effects of DSS-induced colitis in mice and the potential role of EGCG in mitigating these effects, we monitored the body weight of the mice over time. During the preventive EGCG gavage phase, the mice experienced an increase in body weight, but after the treatment, a significant difference was observed. The DSS group showed the lowest body weight, while EGCG treatment led to a significant recovery (*p* < 0.001) (Figure 2A). Similarly, the DSS group exhibited a significant increase in fecal occult blood index, whereas the EGCG-preventive group had a significantly lower fecal occult blood index compared to the DSS group (Figure 2B). Moreover, EGCG effectively restored the organ index disruptions caused by colitis (Figure S1).

In addition to body weight loss, DSS administration led to abnormalities in the colon phenotype and inflammatory response. Specifically, the DSS group exhibited a significant reduction in colon length (*p* < 0.01) (Figure 2C,D), along with pronounced inflammatory cell infiltration and increased protein expression of pro-inflammatory cytokines IL-6, IL-1β, and TNF-α (*p* < 0.01) (Figure 2E,F and Figure S2). However, preventive supplementation with EGCG effectively alleviated these pathological changes, significantly improving colon morphology and reducing inflammatory markers.

### 3.2. EGCG Supplementation Improves Hematological and Iron Parameters in DSS-Induced Colitis

**Figure 3:**
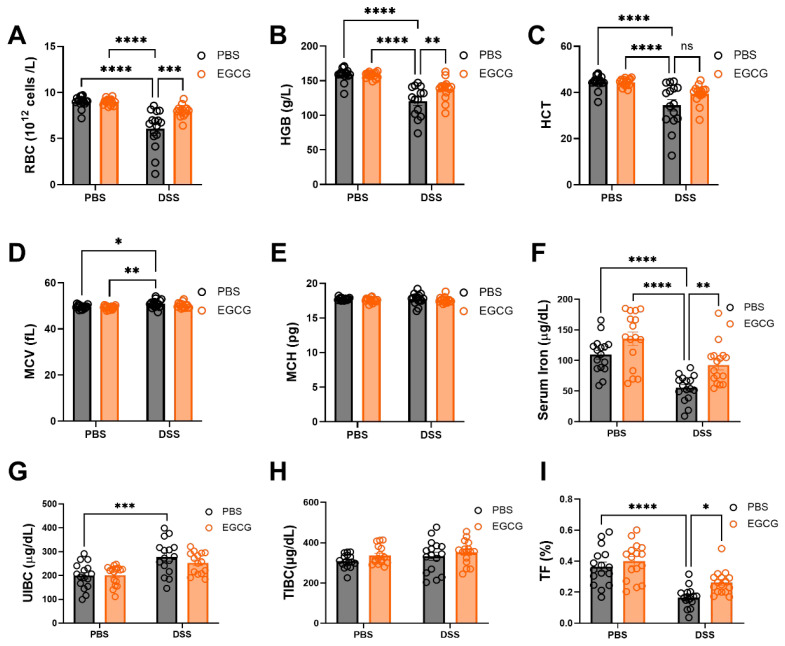
**EGCG seems to contribute to alleviating inflammatory anemia caused by colitis.** (**A**) Red blood cell count (RBC). (**B**) Hemoglobin (HGB) (*n* = 16). (**C**) Hematocrit (HCT) (*n* = 16). (**D**) Mean corpuscular volume (MCV) (*n* = 16). (**E**) Mean corpuscular hemoglobin (MCH) (*n* = 16). (**F**) Serum iron level (*n* = 16). (**G**) Unsaturated iron-binding capacity (UIBC) (*n* = 16). (**H**) Total iron-binding capacity (TIBC) (*n* = 16). (**I**) Transferrin saturation (TF) (*n* = 16). Statistical significance is indicated as follows: * *p* < 0.05, ** *p* < 0.01, *** *p* < 0.001, **** *p* < 0.0001.

The blood routine parameters, including RBC, HCT, HGB, MCV, and MCH, as well as serum iron, UIBC, TIBC, and TF levels, were assessed to evaluate the impact of DSS-induced colitis and the effects of preventive EGCG supplementation (Figure 3). DSS treatment resulted in significant reductions in RBC count, HCT, and HGB levels, indicating the development of anemia, along with a decrease in serum iron, UIBC, and TF levels (Figure 3A–C,F,H,I). The decrease in these parameters reflects a disruption in iron homeostasis, which contributes to the overall inflammatory state. Furthermore, MCV was also reduced in the DSS group (Figure 3D), suggesting altered erythropoiesis and iron deficiency. Interestingly, preventive supplementation with EGCG showed a restorative effect on RBC count, HCT, HGB, serum iron, UIBC, and TF levels, demonstrating the potential of EGCG in mitigating the hematological disturbances induced by DSS-induced colitis (Figure 3A–C,F,H,I). However, no significant changes were observed in MCH and TIBC following EGCG supplementation, indicating that these two parameters may not be as responsive to EGCG intervention under the current experimental conditions (Figure 3E,G). These findings suggest that EGCG supplementation can partially reverse the hematologic and iron metabolism alterations caused by DSS-induced colitis, highlighting its potential as a therapeutic agent for inflammatory conditions associated with iron dysregulation.

### 3.3. Preventive EGCG Supplementation Alleviates Colitis by Regulating Tissue Iron Overload and Iron Metabolism

Analysis of tissue iron content and iron metabolism markers revealed significant changes in response to DSS-induced colitis and preventive EGCG supplementation. DSS challenge caused a notable increase in iron deposition across multiple organs, including the liver, spleen, and kidney, indicating systemic iron dysregulation. However, preventive EGCG supplementation significantly reduced iron accumulation in these organs, highlighting its role in restoring iron homeostasis. Interestingly, no significant changes were observed in colonic iron levels, potentially due to the limited capacity of the colon to sequester excess iron or its rapid utilization in local inflammatory responses.

**Figure 4:**
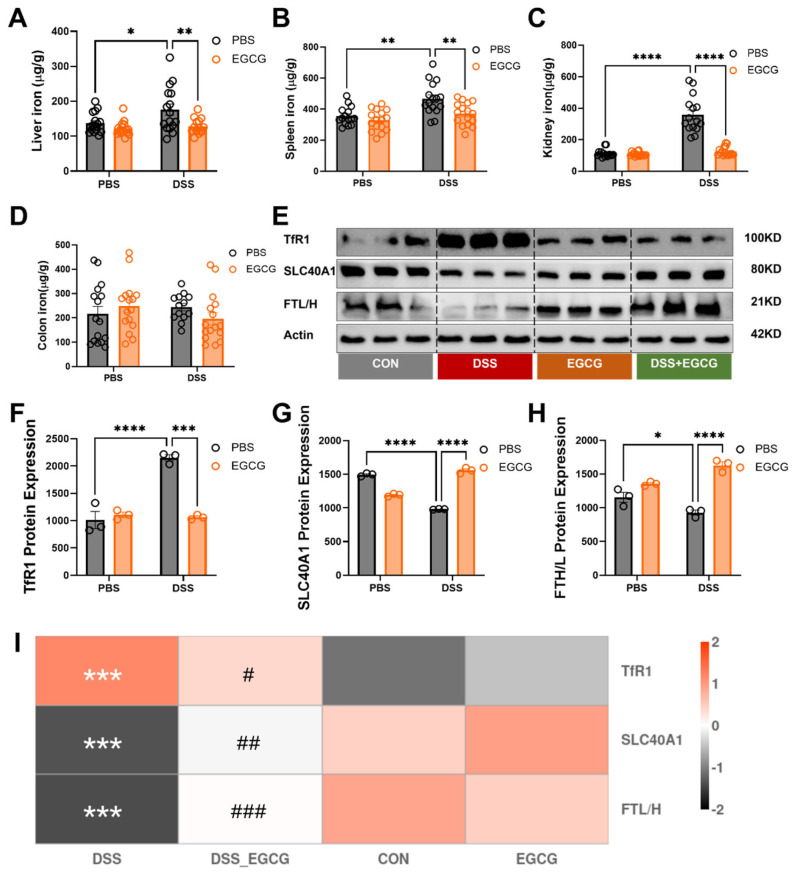
**EGCG alleviates the disrupted iron metabolism caused by colitis.** (**A**) Iron content of the liver (*n* = 16). (**B**) Iron content of the spleen (*n* = 16). (**C**) Iron content of the kidney (*n* = 16). (**D**) Iron content of the colon (*n* = 16). (**E**–**H**) The protein expression level of TfR1, SLC40A1, and FTL in colon of mice (*n* = 3). (**I**) Heatmap of correlation analysis between treatment groups and proteins related to iron metabolism(*^/#^ p < 0.05, **^/##^ p < 0.01, ***^/###^ p < 0.001. * and # represent significance between different treatment groups, with * for DSS vs. CON and # for DSS+EGCG vs. DSS.). Statistical significance is indicated as follows: * *p* < 0.05, ** *p* < 0.01, *** *p* < 0.001, **** *p* < 0.0001.

On the molecular level, DSS treatment led to a marked upregulation of TfR1 and FTH/L at both protein and mRNA levels, consistent with increased cellular iron uptake in response to systemic iron overload. Preventive EGCG supplementation effectively reduced TfR1 and FTH/L expression, reflecting its ability to mitigate iron uptake and accumulation. Furthermore, the expression of ferroportin (Fpn, encoded by SLC40A1), the key iron-export protein, was significantly elevated in the DSS group, likely as a compensatory response to increased iron burden. EGCG supplementation normalized SLC40A1 levels, reducing the excessive iron export and contributing to systemic iron redistribution. These findings indicate that DSS-induced colitis disrupts systemic iron metabolism, leading to aberrant iron accumulation and dysregulation of key iron-related proteins. Preventive supplementation with EGCG alleviates colitis by attenuating tissue iron overload and modulating iron metabolism pathways (Figure 4).

### 3.4. EGCG Supplementation Attenuates Ferroptosis and Restores Redox Balance in DSS-Induced Colitis

**Figure 5:**
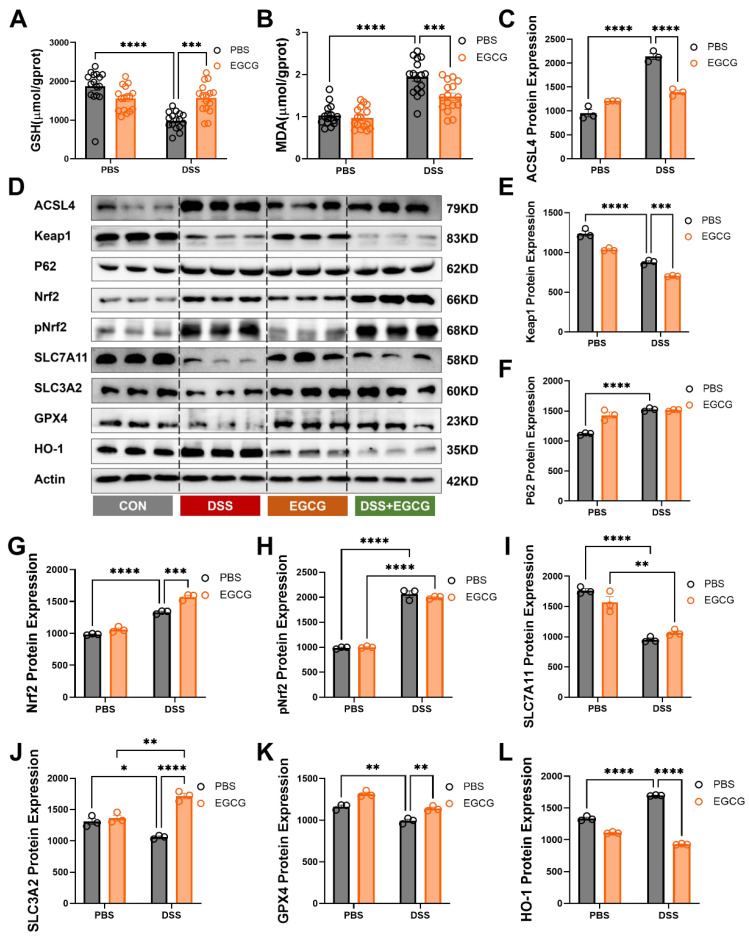
**EGCG alleviates colitis by inhibiting the occurrence of ferroptosis in cells.** (**A**) GSH level in the colon (*n* = 16). (**B**) MDA level in the colon (*n* = 16). (**C**) Quantification of ACSL4 protein expression (*n* = 3). (**D**) Western blot analysis of ferroptosis-related protein expression (*n* = 3). (**E**–**L**) Quantification of Keap1, P62, Nrf2, pNrf2, SLC7A11, SLC3A2, GPX4, and HO-1 protein expression (*n* = 3). Statistical significance is indicated as follows: * *p* < 0.05, ** *p* < 0.01, *** *p* < 0.001, **** *p* < 0.0001.

Preventive EGCG supplementation demonstrated significant regulatory effects on ferroptosis markers and related proteins in DSS-induced colitis. DSS challenge led to a substantial increase in malondialdehyde (MDA), a marker of lipid peroxidation, and a marked depletion of glutathione (GSH), indicating elevated oxidative stress and ferroptosis activation. EGCG supplementation effectively reduced MDA levels and restored GSH content, alleviating oxidative damage (Figure 5A,B). At the molecular level, DSS treatment disrupted the balance of the Nrf2-signaling pathway, leading to increased expression of Nrf2, phosphorylated Nrf2 (pNRF2), and P62, along with a reduction in KEAP1 levels, a key negative regulator of Nrf2. Preventive EGCG supplementation further enhanced the activation of the Nrf2 pathway by upregulating Nrf2, pNrf2, and P62 expression while simultaneously downregulating KEAP1. This regulation contributed to the activation of the antioxidant defense system, effectively mitigating oxidative stress in DSS-induced colitis (Figure 5D,E–H). Similarly, DSS-induced colitis suppressed the expression of key ferroptosis-regulating proteins such as GPX4, SLC7A11, and SLC3A2, while elevating HO-1 levels as a compensatory response to oxidative stress. EGCG supplementation reversed these changes, significantly increasing GPX4, SLC7A11, and SLC3A2 expression and reducing HO-1 levels (Figure 5D,I–L). Furthermore, DSS treatment caused a significant upregulation of ACSL4, a critical enzyme in lipid peroxidation and ferroptosis. EGCG supplementation markedly decreased ACSL4 expression, thereby mitigating ferroptosis and lipid peroxidation (Figure 5C,D). These results highlight EGCG’s therapeutic potential in alleviating ferroptosis and restoring redox homeostasis in DSS-induced colitis by modulating oxidative stress, iron metabolism, and lipid peroxidation pathways.

### 3.5. Preventive EGCG Supplementation Mitigates Ferroptosis and Inflammatory Responses in RSL3-Induced Cell Model

**Figure 6:**
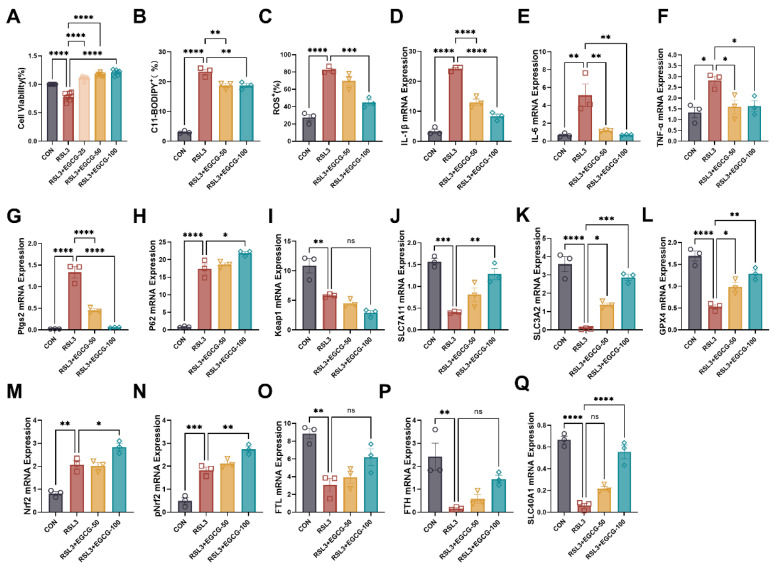
**EGCG inhibits ferroptosis in MCEC.** (**A**) Cell viability (*n* = 6). (**B**) Positive rate of lipid peroxidation in MCEC (*n* = 3). (**C**) Positive rate of total ROS in MCEC (*n* = 3). (**D**–**Q**) IL-1β, IL-6,TNF-α, Ptgs2, P62, Keap1, SLC7A11, SLC3A2, GPX4, Nrf2, pNrf2, FTL, FTH, and SLC40A1 mRNA expression were tested by qPCR (*n* = 3). Statistical significance is indicated as follows: ^ns^ *p* < 0.1, * *p* < 0.05, ** *p* < 0.01, *** *p* < 0.001, **** *p* < 0.0001.

In an RSL3-induced ferroptosis model, significant cellular damage was observed, characterized by reduced cell viability and elevated ferroptosis markers. RSL3 treatment led to a marked decrease in cell viability, as assessed by CCK-8 assays (Figure 6A), accompanied by increased levels of C11-BODIPY, reactive oxygen species (ROS), and intracellular Fe^2+^, indicating enhanced lipid peroxidation and oxidative stress (Figure 6B,C and Figure S3A,B). Preventive EGCG supplementation effectively mitigated these effects, restoring cell viability and reducing the accumulation of ferroptosis markers. At the molecular level, RSL3 treatment induced significant inflammatory responses, as evidenced by elevated gene expression of IL-1β, IL-6, and TNF-α (Figure 6D–F). EGCG supplementation effectively suppressed these inflammatory markers, demonstrating its anti-inflammatory potential in ferroptosis-associated conditions. Furthermore, RSL3 exposure significantly increased the gene expression of PTGS2, Nrf2, pNrf2, and P62, which are associated with ferroptosis and cellular stress responses (Figure 6G,H,M,N). Simultaneously, the gene expression of ferroptosis-regulating proteins, including GPX4, SLC7A11, SLC3A2, and iron metabolism markers such as FTH, FTL, and SLC40A1, was notably downregulated, reflecting disrupted antioxidant defenses and iron homeostasis (Figure 6I–Q). EGCG supplementation reversed these changes by downregulating PTGS2, while upregulating GPX4, SLC7A11, SLC3A2, FTH, FTL, and SLC40A1 gene expression, thereby restoring redox balance and iron metabolism (Figure 6J–L,O–Q). Additionally, EGCG further increased the gene expression of P62, Nrf2, and pNrf2, while the gene expression of Keap1 continued to decrease (Figure 6H,I,M,N). These findings suggest that EGCG mitigates RSL3-induced ferroptosis by modulating oxidative stress, restoring iron metabolism, and reducing inflammatory responses, underscoring its therapeutic potential in ferroptosis-related diseases.

## 4. Discussion

The present study underscores the therapeutic potential of epigallocatechin-3-gallate (EGCG) in mitigating DSS-induced colitis through its multifaceted actions on inflammation, oxidative stress, iron metabolism, and ferroptosis. Beyond its direct effects observed in our experimental models, these findings invite broader insights into the pathophysiological mechanisms of colitis and the potential applicability of EGCG in related diseases.

Colitis, a hallmark of inflammatory bowel diseases (IBD), is driven by chronic immune activation and a breakdown of intestinal barrier function [33]. Inflammatory cytokines such as IL-6, IL-1β, and TNF-α play pivotal roles in exacerbating tissue damage, and oxidative stress further aggravates the inflammatory milieu [34]. Many studies have highlighted the central role of oxidative stress in IBD pathology. For instance, Zeng et al. (2022) reported that elevated ROS levels impair epithelial integrity and activate inflammatory cascades [35]. In our study, DSS-induced colitis resulted in pronounced oxidative damage, reflected by elevated MDA levels and GSH depletion. These findings align with the established notion that oxidative stress is a driver of IBD progression, emphasizing the need for antioxidant-based interventions. EGCG’s ability to restore GSH and reduce MDA levels confirms its potent antioxidant capacity, consistent with prior studies demonstrating EGCG’s role in reducing oxidative stress through Nrf2 activation (Zhang et al., 2020). This suggests that EGCG may act as a dual-function agent, modulating both inflammation and oxidative stress pathways [36].

The disruption of systemic iron metabolism in colitis adds another layer of complexity to the disease mechanism [37]. Iron, a critical cofactor for cellular functions, becomes pathogenic when dysregulated, contributing to oxidative damage and ferroptosis [38]. Iron overload in the liver, spleen, and kidney observed in DSS-treated mice mirrors findings by Swanson et al. (2019), who noted that excessive iron exacerbates inflammatory responses through the Fenton reaction, leading to increased ROS production [39]. Our study demonstrated that EGCG supplementation significantly reduced iron accumulation in these tissues, possibly by regulating key mediators of iron metabolism such as TfR1 and SLC40A1. These findings align with earlier reports showing that EGCG’s iron-chelating properties attenuate hepatic iron overload. Interestingly, we observed no significant changes in colonic iron levels despite systemic dysregulation. This may be attributed to the rapid utilization of free iron in local inflammatory responses, a hypothesis supported by Chen et al. (2024), who noted that colonic epithelial cells prioritize iron utilization for rapid turnover during inflammation [40,41].

One of the most intriguing aspects of this study is the interplay between ferroptosis and colitis [42]. Ferroptosis, a regulated form of cell death driven by iron-dependent lipid peroxidation, has emerged as a central mechanism in inflammatory and degenerative diseases [43]. Increased ACSL4 expression and reduced GPX4 levels observed in our study confirm ferroptosis activation in DSS-treated mice, consistent with the findings of Xu et al. (2020), who demonstrated ferroptosis as a critical contributor to epithelial cell death in IBD [44,45]. EGCG supplementation mitigated these changes, restoring GPX4 and reducing ACSL4 expression, highlighting its potential to regulate ferroptosis through both antioxidant and iron homeostasis pathways [45]. Additionally, the role of Nrf2 signaling in ferroptosis cannot be overlooked. DSS treatment disrupted the Nrf2 pathway by increasing p Nrf2 and P62 while suppressing Keap1 [46,47]. EGCG effectively restored Nrf2 pathway homeostasis, a finding consistent with Sun et al. (2021), who demonstrated that Nrf2 activation protects against ferroptosis by upregulating GPX4 and SLC7A11 [42].

From a translational perspective, the restoration of hematological parameters by EGCG highlights its potential clinical relevance. Anemia of inflammation, a common comorbidity in IBD, is characterized by impaired erythropoiesis and iron sequestration. DSS-treated mice exhibited reduced RBC, HCT, and HGB levels, reflecting these hallmarks. EGCG’s ability to normalize these parameters by modulating iron homeostasis and reducing inflammatory cytokines underscores its potential to address anemia in inflammatory conditions. This is consistent with the work of Ganz (2020), who emphasized the importance of hepcidin-SLC40A1 regulation in anemia of inflammation [13]. By downregulating TfR1 and normalizing SLC40A1 expression, EGCG appears to modulate the Hamp1-SLC40A1 axis, a critical pathway in maintaining systemic iron balance.

Our findings also extend to the role of EGCG in the cellular context, as demonstrated in the RSL3-induced ferroptosis cell model. The reduction of IL-1β, IL-6, and TNF-α by EGCG reflects its anti-inflammatory properties, which are well-documented in the literature [34]. Furthermore, EGCG’s regulation of ferroptosis-related proteins, such as GPX4, SLC7A11, and ACSL4, aligns with its observed effects in vivo, underscoring its dual anti-inflammatory and anti-ferroptosis mechanisms [48]. These observations highlight the potential of EGCG as a therapeutic agent for ferroptosis-related diseases, extending beyond colitis to conditions such as neurodegenerative diseases and cancer, as suggested by Tang et al. (2024) [49,50].

In conclusion, this study reveals the multifaceted mechanisms through which EGCG mitigates DSS-induced colitis, encompassing anti-inflammatory, antioxidant, and ferroptosis-regulatory pathways. Its ability to restore iron homeostasis and suppress ferroptosis highlights its therapeutic potential in inflammatory and ferroptosis-associated diseases. Future research should focus on optimizing EGCG formulations to enhance bioavailability and exploring its synergistic potential with existing therapies to fully harness its clinical utility.

## 5. Conclusions

This study has limitations, including the inability of the DSS-induced colitis model to fully mimic human IBD complexity and a small sample size, which may limit generalizability. Future studies with larger cohorts and alternative models are needed. Despite this, EGCG shows potential as an IBD therapy. Future research should explore its efficacy in humans, optimize dosage and bioavailability, and evaluate its combination with existing treatments. Additionally, investigating EGCG’s role in other inflammatory and iron-related diseases could expand its clinical applications.

## Data Availability

This study was not pre-registered in a publicly accessible protocol repository, as is common in preclinical animal research. Future studies will aim to follow standardized protocol registration practices. The datasets generated and analyzed during the current study are available from the corresponding author upon reasonable request.

## Supplementary Materials

The following supporting information can be downloaded at: https://www.mdpi.com/article/10.3390/nu17030547/s1. Figure S1. EGCG might mitigate the abnormal organ measurements linked to colitis. (A) Liver weight in different treatment groups. (B) Spleen weight in different treatment groups. (C) Kidney weight in different treatment groups. (D) Liver index (liver weight/body weight ratio). (E) Spleen index (liver weight/body weight ratio). (F) Kidney index (liver weight/body weight ratio). Statistical significance is indicated as follows: * *p* < 0.05, ** *p* < 0.01, *** *p* < 0.001, **** *p* < 0.0001. Figure S2. Effects of EGCG on Inflammatory Markers IL-6, IL-1β, and TNF-α in Colitis. (A) Serum IL-1β content. (B) Serum IL-6 content. (C) Serum TNF-α content. Statistical significance is indicated as follows: * *p* < 0.05, ** *p* < 0.01, *** *p* < 0.001, **** *p* < 0.0001. Figure S3. EGCG Inhibits ROS and Fe^2+^ to Alleviate Ferroptosis. (A) Flow cytometry images of lipid peroxidation in MCEC. The cells were stained with 5 μM C11-BODIPY, a dye used to detect lipid peroxidation. Flow cytometry analysis was performed to assess the positive rate of lipid peroxidation in MCEC after treatment with EGCG and RSL3. (B) Flow cytometry histogram of labile iron pool (LIP) in MCEC.

## Abbreviations

**Abbreviations**	**Full Name**
ACSL4	Acyl-CoA synthetase long-chain family member 4
FAC	Ferric citrate
FTH/L	Ferritin heavy/light chain
Fpn	Ferroportin(SLC40A1)
GSH	Glutathione
GPX4	Glutathione peroxidase 4
HGB	Hemoglobin
HCT	Hematocrit
IL-6	Interleukin-6
IL-1β	Interleukin-1β
HO-1	Heme oxygenase 1
Keap1	Kelch-like ECH-associated protein 1
MCV	Mean corpuscular volume
MCH	Mean corpuscular hemoglobin
MCHC	Mean corpuscular hemoglobin concentration
MDA	Malondialdehyde
Nrf2	Nuclear factor erythroid 2-related factor 2
pNrf2	Phosphorylated Nuclear factor erythroid 2-related factor 2
P62	Sequestosome 1
RBC	Red blood cell count
ROS	Reactive oxygen species
SLC3A2	Solute carrier family 3 member 2
SLC7A11	Solute carrier family 7 member 11
TF	Transferrin saturation
TIBC	Total iron binding capacity
TfR1	Transferrin receptor 1
TNF-α	Tumor Necrosis Factor-alpha
UIBC	Unsaturated iron binding capacity
