# Supplementary material for: EGCG Alleviates DSS-Induced Colitis by Inhibiting Ferroptosis Through the Activation of the Nrf2-GPX4 Pathway and Enhancing Iron Metabolism
Source: Nutrients. 2025 Jan 31;17(3):547. doi: 10.3390/nu17030547 (PMC11820173; doi:10.3390/nu17030547)
Supplement: Supplementary file 1 [file nutrients-17-00547-s001.zip › nutrients-3413426-supplementary.pdf]

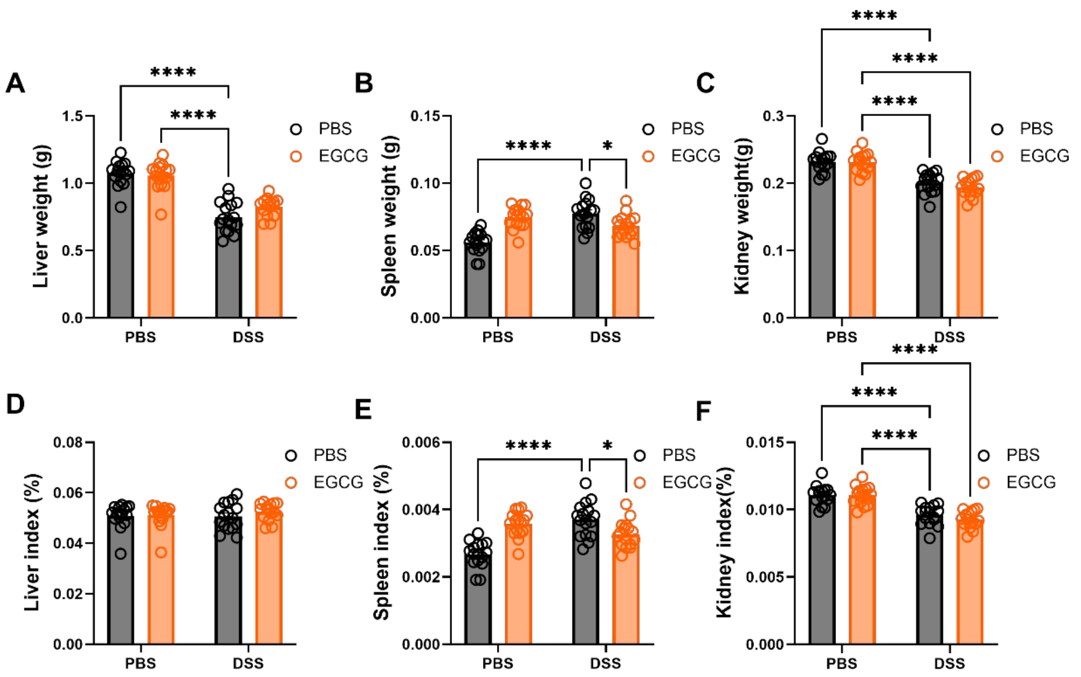

Figure S1. EGCG might mitigate the abnormal organ measurements linked to colitis. (**A**) Liver weight in different treatment groups. (**B**) Spleen weight in different treatment groups. (**C**) Kidney weight in different treatment groups. (**D**) Liver index (liver weight/body weight ratio). (**E**) Spleen index (liver weight/body weight ratio). (**F**) Kidney index (liver weight/body weight ratio). Statistical significance is indicated as follows: * $p < 0.05$, ** $p < 0.01$, *** $p < 0.001$, **** $p < 0.0001$.

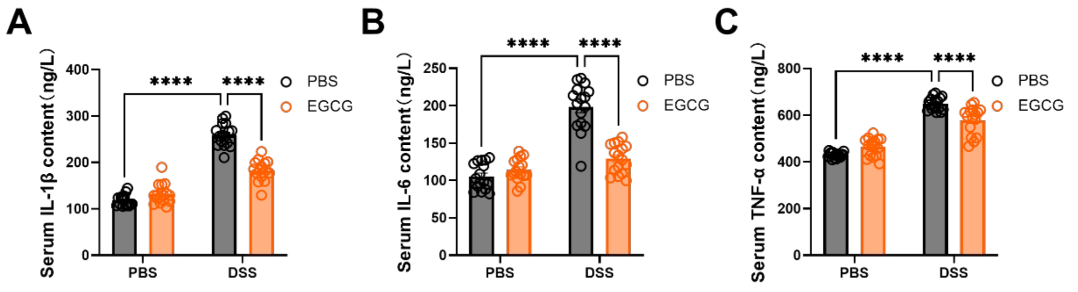

Figure S2. Effects of EGCG on Inflammatory Markers IL-6, IL-1β, and TNF-α in Colitis. (**A**) Serum IL-1β content. (**B**) Serum IL-6 content. (**C**) Serum TNF-α content. Statistical significance is indicated as follows: * $p < 0.05$, ** $p < 0.01$, *** $p < 0.001$, **** $p < 0.0001$.

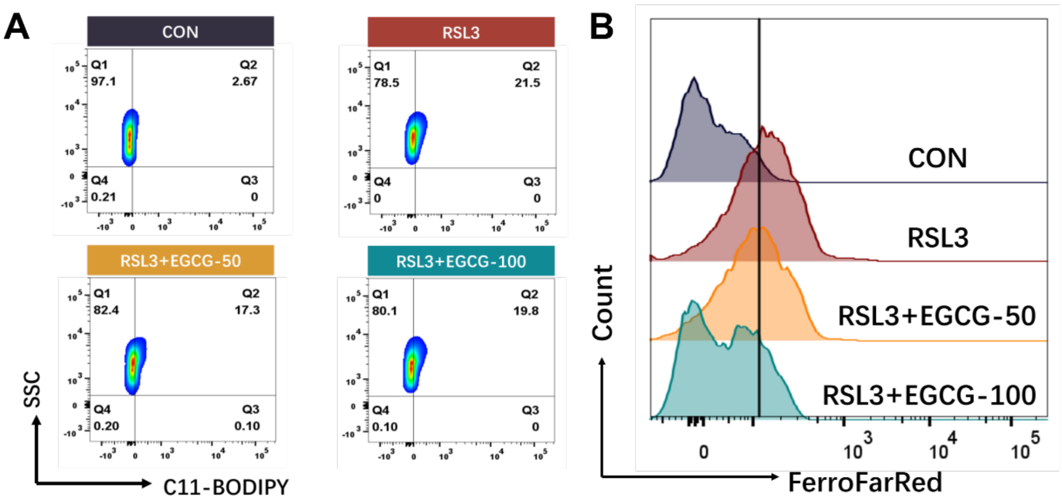

Figure S3. EGCG Inhibits ROS and Fe^{2+} to Alleviate Ferroptosis. (**A**) Flow cytometry images of lipid peroxidation in MCEC. The cells were stained with 5 μM C11-BODIPY, a dye used to detect lipid peroxidation. Flow cytometry analysis was performed to assess the positive rate of lipid peroxidation in MCEC after treatment with EGCG and RSL3. (**B**) Flow cytometry histogram of labile iron pool (LIP) in MCEC.
